# Evaluation of a New Treatment Strategy for Geriatric Fragility Fractures of the Posterior Pelvic Ring Using Sensor-Supported Insoles: A Proof-of-Concept Study

**DOI:** 10.3390/jcm12165199

**Published:** 2023-08-10

**Authors:** Luca Lebert, Alexander Martin Keppler, Jan Bruder, Leon Faust, Christopher Alexander Becker, Wolfgang Böcker, Carl Neuerburg, Adrian Cavalcanti Kußmaul

**Affiliations:** Department of Orthopaedics and Trauma Surgery, Musculoskeletal University Center Munich (MUM), LMU University Hospital, LMU Munich, Marchioninistraße 15, 81377 Munich, Germany

**Keywords:** fragility fractures of the pelvis, orthogeriatric, sacral fractures, trauma surgery, iFuse Implant System^®^

## Abstract

The incidence of fragility fractures of the pelvis (FFPs) is currently rising. Surgical treatment, which is performed using sacroiliac screws, is complicated by compromised bone quality, oftentimes resulting in implant failure. The iFuse implant system aims to improve attachment and durability with promising results for sacroiliac dysfunction, though data for its feasibility on FFPs are rare. Consequently, this study aims to evaluate the feasibility of the iFuse for FFPs. A total of 10 patients with FFPs were treated with the iFuse in this study. Pre- and postoperatively, both mobility using an established insole force sensor for an inpatient gait analysis and general well-being and pain using questionnaires were evaluated. When comparing pre- and postoperative findings, this study demonstrated a significant increase in the average (8.14%) and maximum (9.4%) loading (*p* < 0.001), a reduction in pain, as measured by the visual analog scale (VAS), from 4.60 to 2.80 at rest (*p* = 0.011) and from 7.00 to 4.40 during movement (*p* = 0.008), an increase in the Barthel Index by 20 points (*p* < 0.001) and an increase in the Parker Mobility Score by 2.00 points (*p* = 0.011). All this contributes to the possibility of early postoperative mobilization and improved general well-being, ultimately preventing the late consequences of postoperative immobilization and maintaining patients autonomy and contentment.

## 1. Introduction

An increase in life expectancy due to the current demographic change and an improvement in quality and access to medical care have led to an extended mobility demand in the aging society [1]. Yet an increasing age also bears the risk of osteopenia and osteoporosis, which can be aggravated due to long-term immobilization or vitamin D deficiency [2,3]. The compromised bone quality also favors geriatric patients suffering from fragility fractures of the pelvis (FFPs), which encompass two entities: fractures after low-energy trauma and pelvic insufficiency fractures without trauma [4]. These fractures are characterized by an exclusive osseous fracture without rupture of the adjacent ligaments, as occurs in high-impact trauma [2]. According to the World Health Organization (WHO), a fragility fracture is caused by an inadequate injury as a result of reduced compressive and/or torsional strength of the bone [5]. Clinically, patients report immobilizing pain in the pubic region, groin and lower back after a fall from a sitting or standing position [6]. Due to the decrease in bone density, the anterior ala ossis sacri is most frequently fractured when observing FFPs [7]. If radiographic imaging in an anterior–posterior (a.p.) inlet and outlet view is inconspicuous due to absent dislocation, computed tomography (CT) or magnetic resonance imaging (MRI) is indicated for adequate diagnostic management [6].

Furthermore, Rommens and Hofmann developed a now-established classification system for FFPs that additionally takes into account the morphology of the fracture and the degree of instability: Mild instability describes an FFP type I that is limited to the anterior pelvic ring. An FFP type II is characterized by a non-dislocated fracture of either the posterior pelvic ring only (FFP IIa), a compression of the sacral massa lateralis combined with an instability of the anterior pelvic ring (FFP IIb) or a non-dislocated complete lesion of the posterior pelvic ring combined with an anterior instability (FFP IIc) [2]. An FFP type III is a unilateral, dislocated posterior injury with anterior instability, while an FFP type IV includes a complete dissociation between the lumbar spine and the pelvic ring, consisting of a bilateral and complete dorsal rupture combined with different morphologies of a unilateral or bilateral anterior rupture [6].

Considering the treatment of FFPs, the ultimate goal lies in the retrieval of the highest mobility possible with a concomitant maximization of individual independence. This can only be achieved by adequate pain relief and early mobilization, as prolonged immobilization of patients due to pain only promotes osteoporosis and sarcopenia, leading to patient bed confinement [3,5]. For FFPs classified as type II or higher, conservative treatment is no longer sufficient based on both the immediate instability potentially causing severe pain and the risk of increasing instability or non-union after early mobilization [6]. When indicated, different surgical techniques can be considered depending on the FFP type: The most common one consists of one or two sacroiliac screws (SI screws), which are used for the stabilization of the ala ossis sacri of the posterior pelvic ring in FFP types II, III and IV. Although this minimally invasive surgery intends to minimize blood loss and perioperative complications, the biggest issue with SI screws regarding their application in FFPs lies in the compromised bone quality available for screw fixation, resulting in lower screw stability and the potential of recurrent or even progressive instability of the posterior pelvic ring [5,6].

To address this problem, SI-BONE^TM^ (Santa Clara, CA, USA) has developed the iFuse Implant System^®^ (iFuse). With its triangular profile and surface of porous titanium, the implant mimics the natural cancellous or trabecular bone structure [8,9]. The fenestrated design additionally allows improved attachment and ingrowth into the bone, consequently offering high resistance to shear and bending loads and a 31× greater resistance to rotation compared to a 7.3 mm screw [8,10]. In addition, the porous surface allows the self-extraction of bone material during implant placement [9]. Furthermore, the minimally invasive surgical procedure with only a small skin incision results in little soft tissue injury and blood loss, which itself reduces peri- and postoperative complications [11,12,13]. To prevent the ablation of soft tissue when inserting the iFuse, a cannulated insertion system and protection instruments for the surrounding tissue are used [14]. Therefore, although the iFuse was originally designed for the treatment of degenerative SI joint diseases, the implant appears to provide a promising approach in trauma cases with reduced bone stock and subsequent fractures in the posterior part of the pelvic ring.

Generally, the implants are available in lengths of 30–90 mm in steps of 5 mm, whereas the outer diameters range between 8.2 and 14.2 mm [14].

Consequently, the aim of this study was to compare pre- and postoperative mobilization after surgical treatment of FFP types II, III and IV with the iFuse using sensor-supported insoles (Loadsols^®^, Novel, Munich, Germany). Previous studies have already demonstrated the effectiveness of sensor-supported insoles for the detection of compromised mobility [15,16].

Also, the authors investigated the weight-bearing ability of the lower extremity after FFP treatment with the iFuse within a few days of the operation and if pain and subjective general well-being were affected. A faster and more effective mobilization could eventually prevent the progression of sarcopenia and osteoporosis and therefore optimize the outcome of geriatric patients with fragility fractures of the pelvis.

## 2. Materials and Methods

### 2.1. Participants and Treatment Principles

**Figure 1:**
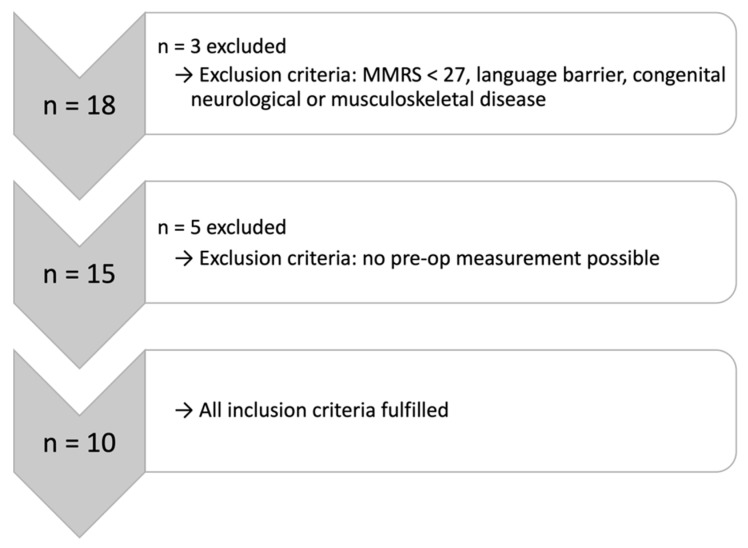
Selection of the study population.

Patients aged 65 years and older suffering from FFP types II–IV according to Rommens et al. between December 2021 and April 2023 were included in this study [6]. Exclusion criteria included a Minimal Mental State Exam (MMSE) score of less than 27, a language barrier, an additional fracture and a congenital neurological or musculoskeletal disease that distorts the gait pattern on the forearm walker (Figure 1).

**Figure 2:**
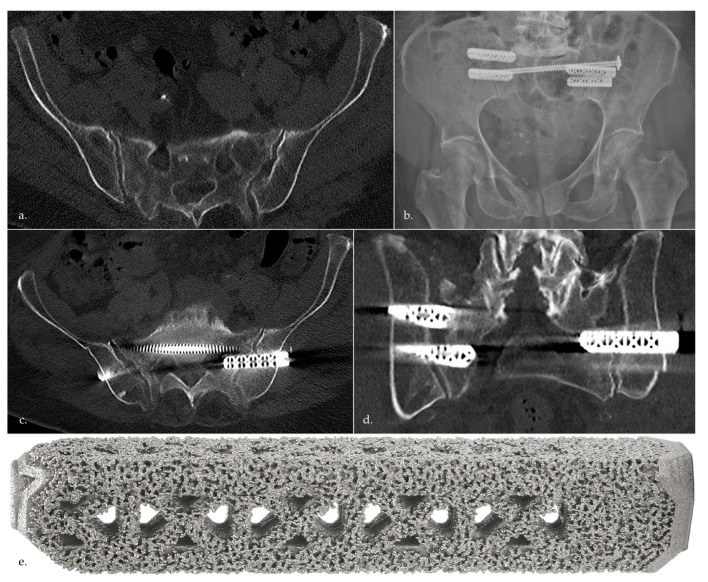
Example of a patient suffering from FFP treated with iFuse and TIS. (**a**) FFP IVc (bilateral massa lateralis fracture); (**b**) postoperative X-ray; (**c**,**d**) postoperative axial and coronal CT scan for visualization of implant placement; (**e**) exemplary illustration of an iFuse Implant System^®^.

Initially and after a computertomography (CT) scan, the fractures were classified according to the FFP classification by two senior physicians and additionally confirmed by the surgeon [6]. After presentation of patients at our level I trauma center with immobilizing and persistent lower back pain after failed conservative treatment, surgical stabilization of an FFP type II using iFuse combined with transiliosacral screws (TISs) (TIS^TM^ screws, Königsee Implantate GmbH, Allendorf, Germany) was performed. Conservative treatment was considered failed when patients still complained of being insufficiently mobile after four to seven days. In the occurrence of an FFP type IV, surgery was performed immediately due to the instability of the pelvic ring. Patients with an FFP type II only received stabilization of the posterior pelvic ring using at least two iFuse insertions and one TIS unilaterally on the fractured site. Patients with an FFP type IV received supplementary osteosynthesis (retrograde screw fixation or angle-stable plate osteosynthesis) of the anterior pelvic ring fracture in addition to the use of a minimum of two iFuse insertions placed bilaterally and one TIS spanning through both sacral alae for the posterior pelvic ring (Figure 2). No patient suffered from an FFP type III.

In detail, the iFuse insertion is similar to the insertion of a TIS using a guidewire, which is placed transiliosacrally in the S1 or S2 channel either under fluoroscopic control or with navigation. Yet the iFuse is not screwed in but gently hammered in with the appropriate instrumentation.

Additionally, a pre- and postoperative examination using sensor-assisted insoles (Loadsols^®^, Novel, Munich, Germany) was performed. The postoperative examination took place within four to seven days of surgery.

### 2.2. Study Design

This study represents a prospective observational clinical study. Approval from the ethics committee (approval no. 214-16) was obtained prior to this study. Also, the written informed consent of all participants was gathered in advance. An a priori power analysis was performed using G*Power 3.1 (Heinrich Heine University, Düsseldorf, Germany). To reach a power of 80% at a significance level of *p* < 0.05, a necessary sample size of 10 patients was determined.

### 2.3. Gait Analysis

**Figure 3:**
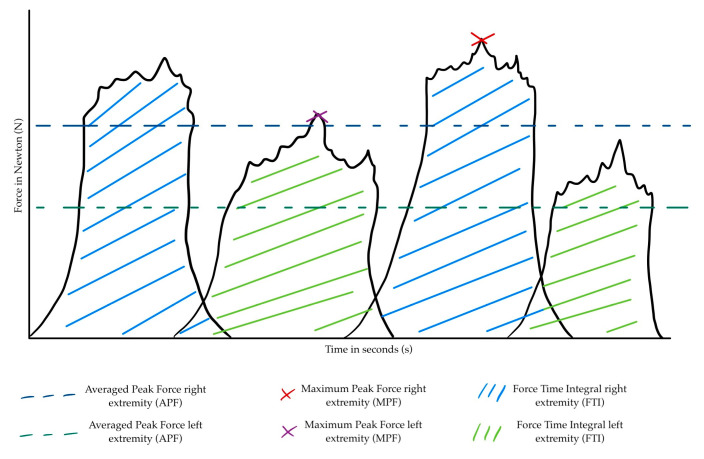
Graphical representation of the collected parameters.

To measure the difference between pre- and postoperative mobilization, sensor-assisted insoles were used. The insoles use a sensor to measure the general force transmission of the body to the soles of the shoes, especially the load on the respective lower extremity. Therefore, the insoles were first inserted into the shoes. Then, the insoles were connected to the hardware via Bluetooth, for which a mobile iPad (Apple Inc., Cupertino, CA, USA) was used. Afterwards, the patients were asked to walk a predefined distance of at least 20 m on the forearm walker. The mobile hardware assesses the gait pattern in real time. The exact analysis of the parameters is then carried out using the loadsol app (Version 1.4.72., Novel, Munich, Germany) (Figure 3). Patients were asked about their pain at rest while lying in bed and during movement (during the gait analysis) both before and after surgical treatment using the visual analog scale (VAS). At the time of measurement, all patients were provided with adequate pain medication according to the WHO (VAS ≤ 4), and no patient had a local pain catheter.

The Force Time Integral (FTI) is the total load (N) of the respective limb over the entire measurement period (s). It is equivalent to the area under the curve (AUC). The strength and length of the load on the limb corresponded proportionally to the FTI, from which the FTI ratio was subsequently calculated. This ratio describes the load balance between the right and left extremities. An FTI ratio of 50% means an optimal force distribution between the right and left lower extremities with an optimal gait balance, while a value of 100% represents a maximum uneven force distribution with one limb being loaded 100% and the contralateral limb being not loaded at all. With the Averaged Peak Force (APF) (N), the maximum force value of each step was added and then divided by the number of steps, resulting in the average. It thus describes the average load on the individual lower extremities. To be able to compare the patients, the APF (N) was divided by the patient’s body weight in Newton, which results in the corrected APF. The Maximum Peak Force (MPF) indicates the maximum force value for the left and right extremities. For this purpose, the graph with the highest peak is selected for both extremities and measured. Subsequently, the MPF is also indicated as a percentage of the respective body weight.

### 2.4. Clinical Questionnaires

In addition, each patient preoperatively received a standardized survey questionnaire that included the Barthel Index (BI), the Parker Mobility Score (PMS), the EQ-5D, the Minimal Mental State Examination (MMSE) and the Charlson Comorbidity Index (CCI). Postoperatively, patients were only queried using the BI, PMS and EQ-5D. In detail, the BI assesses areas of basic everyday functions, such as using the toilet, climbing stairs and performing personal hygiene [17]. Complex activities, such as going shopping, are determined using the PMS [18]. Here, movement in the house was equated with movement in the wardroom, movement outside the house with movement in the ward and going shopping with leaving the ward. Values between 0 and 100 can be achieved for the BI and values between 0 and 9 for the PMS [17,18]. The MMSE is a standardized dementia test with a score between 0 and 30 [19]. While scores between 28 and 30 are normal, scores below 27 are considered mild cognitive impairment and below 24, mild dementia [19]. The CCI describes the mortality of patients based on previous diseases (heart attack, systolic heart failure, chronic lung disease, etc.) and age, while the EQ-5D assesses the patient’s quality of life using the 5 dimensions (mobility, self-care, activities of daily living, pain/discomfort and anxiety/depression) with a range of 0 to 100 [20,21].

### 2.5. Statistical Analysis

APF, MPF and FTI ratio were measured both pre- and postoperatively. Thus, this study contains a dependent or linked sample, as the two groups consist of the same patients. The data were tested for normal distribution using the Kolmogorov–Smirnov and Shapiro–Wilk tests. To compare the mean values of the normally distributed data sets, the *t*-test for paired samples was carried out afterwards, and the Wilcoxon test was used for the distribution-free data sets. These tests evaluate whether the mean values of two dependent variables are different. The strength of the relationship between the variables (closeness and direction of the correlation) was tested with the bivariate Pearson correlation. The significance level was set to *p* < 0.05. The statistical analysis was performed using IBM SPSS Statistics version 29 (IBM Deutschland GmbH, Ehingen, Germany). A graphic illustration was also created using IBM SPSS Statistics Version 29 (IBM Deutschland GmbH, Ehingen, Germany).

## 3. Results

### 3.1. Demographics of the Participants

A total of 10 patients fulfilled the required criteria and were able to complete the gait analysis according to the study protocol. Altogether, 90% of all patients were women (9/10) and 10% were men (1/10), with a median age of 80.30 years (SD ± 3.77 years, overall range 74–87 years). Participants had an average weight of 60.00 kg (SD ± 11.40 kg, overall range 43–75 kg) and an average BMI of 22.47 kg/m^2^ (SD ± 3.98 kg/m^2^, overall range 15.59–27.29 kg/m^2^). The majority of patients suffered from an FFP type IIb (*n* = 5, 50%), followed by an FFP type IIc (*n* = 3, 30%) and an FFP type IVc (*n* = 2, 20%). There was no FFP type III.

### 3.2. Results of the Questionnaires

Patients scored an average of 29.60 points (interquartile range (IQR) 29–30) on the MMST and 4.40 points (IQR 3.75–5.25) on the CCI. Furthermore, the BI improved from 50.00 points (SD ± 12.91) preoperatively to 70.00 points (SD ± 9.72) postoperatively (*p* < 0.001), while the PMS increased from 1.89 points (SD ± 1.17) to 3.89 points (SD ± 0.78) (*p* = 0.011). The EQ-5D also increased from 26.00 points (SD ± 14.30) to 51.00 points (SD ± 13.70) postoperatively (*p* < 0.001) (Table 1).

**Figure 4:**
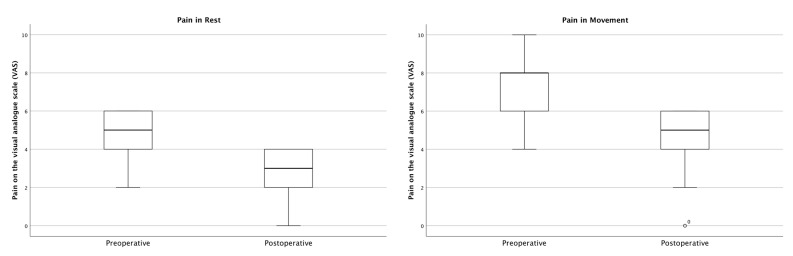
Comparison of pre- and postoperative pain.

The pain at rest decreased from VAS 4.60 (SD ± 1.65) to VAS 2.80 (SD ± 1.40) (*p* = 0.011), while the pain during movement decreased from VAS 7.00 (SD ± 1.94) to VAS 4.40 (SD ± 2.10) (*p* = 0.008) (Figure 4).

### 3.3. Gait Analysis Results

**Figure 5:**
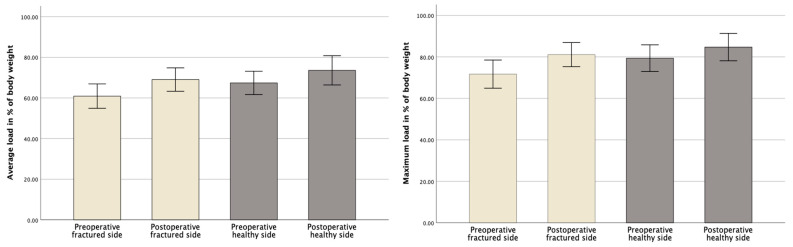
Comparison of pre- and postoperative average and maximum load.

In general, the fractured side was loaded less in the average and maximum loads, both pre- and postoperatively (Figure 5). After surgical treatment with the iFuse, an overall increase in the average and maximum loading could be seen. This increase in loading was more distinct on the fractured pelvic side, resulting in a synchronization of the loading on the two extremities.

**Figure 6:**
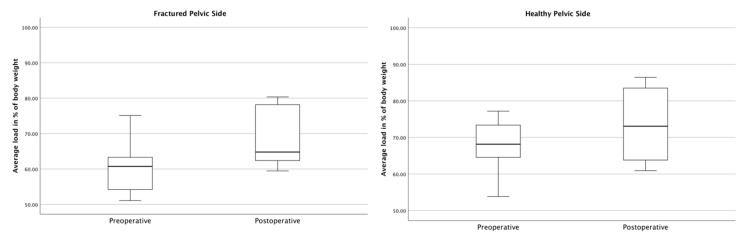
Comparison of pre- and postoperative average loads on the fractured and healthy pelvic sides.

In detail and as displayed in Figure 6, the loading on the fractured side increased significantly on the 4th–7th postoperative days. Before surgery, the average loading on the fractured side was 60.90% (SD ± 8.38%) of the body weight. Postoperatively, the average loading on the operated pelvic side increased to 69.04% (SD ± 8.06%) of the body weight (*p* < 0.001). The average loading of the contralateral, healthy pelvic side also increased from 67.40% (SD ± 8.05%) to 73.60% (SD ± 10.06%) of the body weight (*p* = 0.009).

**Figure 7:**
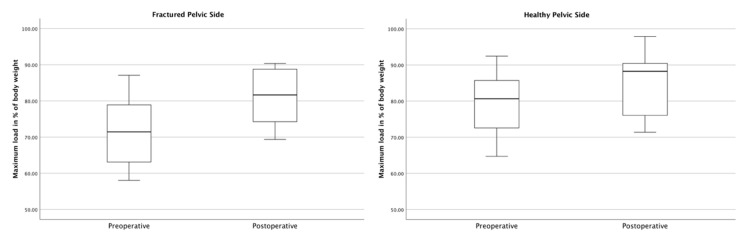
Comparison of pre- and postoperative maximum loads on the fractured and healthy pelvic sides.

Regarding the maximum loading, the patients’ maximum loading also increased significantly postoperatively. While the patients loaded the fractured pelvic side with an average maximum of 71.70% (SD ± 10.06%) of their body weight before surgery, this value increased to 81.10% (SD ± 8.13%) of their body weight after surgery (*p* < 0.001) (Figure 7). The maximum loading on the contralateral, healthy pelvic side was 79.36% (SD ± 9.02%) of their body weight preoperatively and 84.73% (SD ± 9.24%) postoperatively (*p* = 0.011).

**Figure 8:**
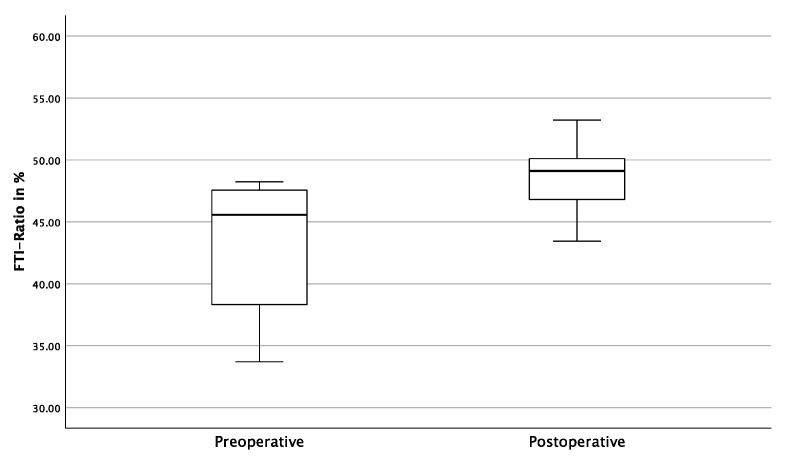
Comparison of pre- and postoperative FTI ratios.

Regarding the FTI ratio, patients displayed an average FTI ratio of 43.35% (SD ± 5.60) preoperatively, demonstrating less loading of the fractured pelvic side during the gait analysis. Between the 4th and 7th days after surgical treatment, the FTI ratio improved to 48.81% (SD ± 2.79%), approaching an almost optimally synchronized gait pattern (*p* = 0.005) (Figure 8).

## 4. Discussion

The incidence of fragility fractures of the pelvis is constantly increasing based on current demographic changes and the concomitant rise in the elderly population [2,4,22,23].

Sterneder et al., for example, observed an increase in insufficiency fractures without trauma compared to low-energy pelvic ring fractures from 5.0% in 2009 to 17.8% in 2017 [4]. Studies from Belgium and Finland also confirm an increase in geriatric pelvic ring fractures. While the overall incidence in Belgium increased from 15.8/100,000 in 1988 to 37.6/100,000 in 2018, the incidence in Finland increased from 73/100,000 in 1970 to 364/100,000 in 2013 [22,23]. In Germany, the current incidence of geriatric pelvic ring fractures in patients aged over 60 years is 224/100,000 [2]. Also, FFPs are associated with a significant increase in morbidity and mortality. While Andrich et al. reported a one-year mortality rate between 8 and 27% [24], Rommens et al. found an overall one-year mortality rate between 16.7% and 22.2% in the patient group over 80 years [25]. In addition, the study found a decrease in the proportion of surviving patients living at home with or without assistance from 80.5% to 65.3% after suffering from an FFP [25]. To outline the significance of these data, the reference value for the one-year mortality of the general population at that age is indicated to be between 4.0% and 5.9% [25]. These data stress not only the clinical relevance of adequate treatment for insufficiency fractures aiming at the restoration of original mobility but also the socioeconomic significance of this rapidly increasing fracture entity.

An aging society and the general optimization of geriatric care are also associated with higher demands on mobility requirements [1]. FFPs are commonly treated with SI screws, yet based on compromised bone quality, the risk of screw loosening with the consequent loss of mobilization and patient autonomy is significant [5,6]. To address this issue, the iFuse, with its triangular shape and its surface mimicking natural bone structure and therefore providing a greater holding capacity, has already been successfully used for sacroiliac dysfunction syndrome [8,9,10,26,27].

SID causes sacroiliac joint pain in the lower back due to abnormal movements in this area, usually associated with inflammatory processes [8]. Clinical evidence for the iFuse suggests an improvement in pain, the Oswestry Disability Index (ODI) and quality of life, measured using the European Quality of Life 5 Dimensions 3 Level Version (EQ-5D) and the Short Form 36 Health Survey (SF-36) [26]. Bornemann et al. confirmed these findings by investigating 24 patients both preoperatively and 3, 6 and 12 months after surgery of a SID with the iFuse; 15 patients (63%) reported they were no longer taking analgesics after 12 months [8]. A meta-analysis of 20 studies also found that the iFuse has significantly better outcomes considering pain, disability (ODI) and quality of life (SF-36) compared to conventional screw techniques [27].

Yet data for the use of the iFuse in FFPs are rare, which is why this study aims to evaluate pre- and postoperative mobilization and general well-being after surgical treatment with the iFuse in patients with FFP.

The results of this study show that patients loading was significantly higher in the gait analysis four to seven days after surgery using the iFuse. While the average loading on the fractured pelvic side demonstrated a postoperative increase of 8.14% when compared to preoperative levels (*p* < 0.001), the maximum recorded loading on the fractured pelvic side also increased by 9.40% of the body weight (*p* < 0.001) (Figure 6 and Figure 7). Taking into account the slight increase (APF + 6.20% (*p* = 0.009); MPF + 5.37% (*p* = 0.011)) on the contralateral, healthy pelvic side, both the average and maximum loadings on both sides approach equilibrium. This reflects an almost synchronous postoperative gait pattern, which is further underlined by a postoperative FTI ratio increase from 43.35% to 48.81% (*p* = 0.005) (Figure 8). This overall increase in postoperative weight-bearing could be a decisive benefit, especially for geriatric patients suffering from osteoporosis and sarcopenia, as these results suggest the potential of a faster and more effective mobilization with a concomitant decrease in bone and soft tissue loss.

Furthermore, one of the principal goals for the treatment of FFPs lies in the reduction in pain, which significantly impacts patients’ mobility. Several studies have compared postoperative pain after treatment of sacroiliac pain syndrome using sacroiliac screws and iFuse implants: A meta-analysis of 20 studies showed that the average pain of patients after surgical treatment with sacroiliac screws was 2.04 on the VAS scale, while the iFuse group had a score of 1.28 [27]. Another study confirmed the results with a significant and immediate reduction in pain from VAS 8.43 to 4.07 after surgery with the iFuse for sacroiliac pain syndrome [28]. After three months, pain even improved to VAS 2.77 and then remained constant between VAS 2.77 and 2.65 for the next few months [28]. Consequently, this study was able to confirm these results for the treatment of FFPs with the iFuse, as displayed by the reduction in pain at rest from VAS 4.60 to 2.80 (*p* = 0.011) and during movement from 7.00 to 4.40 (*p* = 0.008) (Figure 4). This again underlines the possibility of earlier mobilization of patients suffering from FFPs treated with the iFuse.

Furthermore, the reduction in muscle mass in geriatric patients due to immobilization promotes the progression of osteoporosis as mechanical forces acting on the bones through muscle contractions affect bone density, strength and microarchitecture [29]. For this reason, a decrease in muscle mass leads to a consequent loss of bone mass, which further deteriorates bone quality. In addition, sarcopenia leads to a decrease in muscular stability, which can increase the incidence of falls and elevate the risk of new fractures [30]. Here, the treatment of FFPs with the iFuse improved both the intensity and synchrony of loading and pain. This overall potential for fast and effective mobilization can therefore presumably reduce muscle atrophy and the associated progression of osteoporosis.

Also, a standardized treatment protocol is necessary to prevent long-term immobilization based on inconclusive decision-making. Studies show that overall mortality after FFP types II to IV is significantly lower in patients receiving surgical treatment [31]. In addition, a faster preoperative decision-making process is associated with a shorter hospital stay [32]. Geriatric patients thus massively benefit from early mobilization, as mentioned above, which is why adequate, stable fracture fixation is necessary. Yet as osteoporotic bone offers little substance for screw fixation, conventional SI screws are oftentimes inadequate and associated with a high failure rate [33]. This screw loosening is mostly the result of the movement of not only the sacral fracture but also the SI joint. Here, the iFuse, with its unique structure and surface, aims to provide a more stable fixation in the bone and also ensure immobilization, especially regarding rotation in the frontal axis of the SI joint [8,9,10]. In a prospective study regarding the revision rate of sacroiliac joint fusions with the iFuse, the revision rate after 18 months was 2.8% (*n* = 172), while another study found a revision rate of 0.98% (*n* = 102) [34,35]. The rate even decreases with increasing surgeon experience (>100 procedures) to 0.74% [36]. To verify whether the revision rate after surgical treatment of FFPs with iFuse implants is equally low, further studies with follow-up examinations of patients need to be conducted.

This study also showed that the observed increase in postoperative loading also corresponded positively with the subjective feelings of the patients (Table 1). The Barthel Index increased by 20.00 points just a few days after the surgical treatment (*p* < 0.001). Patients also reported an average increase of 2.00 points on the Parker Mobility Score (*p* = 0.011). Therefore, this study did not only demonstrate the improvement in patients’ postoperative biomechanical mobility but also proved the enhancement of patients’ overall postoperative well-being.

Regarding the limitations of this study, the main reason for the limited number of participants was that patients did not fulfill the inclusion criteria based on pain, immobilization or a lack of motivation. Furthermore, this study included a geriatric patient population with many comorbidities, hence, preexisting cognitive and motoric limitations also led to the exclusion of participation in this study based on potential compliance issues. Therefore, the number of participants is currently too low to allow a comparison of the significance of the results to an established treatment method with much more investigated data in the literature. Consequently, no conclusion regarding comparative effectiveness can be drawn. For this reason, this manuscript is intended to be a proof-of-concept study that displays the assumingly positive effect of the iFuse on FFPs a few days after surgery. Accordingly, these findings were not intended to be left unmentioned, as they are very auspicious, laying the foundation for both this proof-of-concept study and further research regarding the use of the iFuse for FFPs.

Moreover, the small patient collective made it neither possible to differentiate between the individual FFP classifications in this study nor to randomize patients, leading to the concomitant potential of an unconscious selection bias. Furthermore, patients were only measured selectively twice, namely before and between four and seven days after surgery. Even though this is most likely the most decisive period when it comes to the question of early inpatient mobilization, no statement can be made regarding long-term mobilization.

Also, the largest number of patients suffered from an FFP type II (50%), while patients with an FFP type III (0%) were not present. This distribution was, however, also noted in other studies [16].

In addition, as this study was performed in a clinical setting, each patient had to overcome different obstacles (e.g., room doors, tight turnings, medical equipment) on the ward during gait analysis, potentially influencing the gait pattern. Also, only limited parameters can be recorded with the insoles. To perform further, more detailed gait analyses, several other wearable motion sensors are available and could be used for evaluation in further studies. Further studies are necessary to assess the feasibility and effectiveness of the iFuse for FFPs, preferably based on a comparison between the iFuse and the conventional SI screw approach.

## 5. Conclusions

This proof-of-concept study indicates that geriatric patients with FFPs who were treated with a combination of the iFuse and SI screws displayed a higher postoperative loading on both lower extremities and achieved a more synchronous gait pattern than preoperatively. Also, preoperative pain was significantly reduced, and the patients’ subjective perception of mobility and quality of life improved in this case series. This overall potential for fast and effective mobilization can therefore presumably reduce muscle atrophy and the associated progression of osteoporosis.

## Figures and Tables

**Table 1:** Comparison of subjective mobility using the Barthel Index, Parker Mobility Score and EQ-5D pre- and postoperatively.

	Preoperative	Postoperative	*p*-Value
Barthel Index	50.00 ± 12.91	70.00 ± 9.72	*p* < 0.001
Parker Mobility Score	1.89 ± 1.17	3.89 ± 0.78	*p* = 0.011
EQ-5D	26.00 ± 14.30	51.00 ± 13.70	*p* < 0.001

## Data Availability

The data presented in this study are available on request from the corresponding author.
